# Evaluation of efficacy and safety for recombinant human adenovirus-p53 in the control of the malignant pleural effusions via thoracic perfusion

**DOI:** 10.1038/srep39355

**Published:** 2016-12-15

**Authors:** Rong Biaoxue, Pan Hui, Gao Wenlong, Yang Shuanying

**Affiliations:** 1 Department of Respiratory Medicine, The First Affiliated Hospital, Xi’an Medical University, Xi’an, China; 2 Research Center of Prevention and Treatment of Respiratory Disease, Shaanxi Province, 710077, Xi’an, China; 3 Department of Respiratory Medicine, Gansu Provincial Hospital, Lanzhou, China; 4 Institute of Epidemiology and Health Statistics, School of Public Health, Lanzhou University, Lanzhou, China; 5 Department of Respiratory Medicine, Second Affiliated Hospital, Xi’an Jiaotong University, Xi’an, China

## Abstract

A certain number of studies have showed that p53 gene transfer has an anti-tumor activity *in vitro* and *in vivo*. This study was to evaluate the efficacy and safety of thoracic perfusion of recombinant human adenovirus p53 (rAd-p53, Gendicine) for controlling malignant pleural effusion (MPE). We searched for the relevant studies from the database of MEDLINE, Web of Science, EMBASE, Cochrance Library and CNKI to collect the trials concerning the efficacy and safety of rAd-p53 to treat MPE. Fourteen randomised controlled trials (RCTs) with 879 patients were involved in this analysis. The rAd-p53 combined with chemotherapeutic agents significantly improved the overall response rate (ORR) (P < 0.001; odds ratio = 3.73) and disease control rate (DCR) (P < 0.001; odds ratio = 2.32) of patients with MPE as well as the quality of life (QOL) of patients (P < 0.001; odds ratio = 4.27), compared with that of chemotherapeutic agents alone. In addition, the participation of rAd-p53 did not have an obvious impact on the most of incidence of adverse reactions (AEs) (P < 0.05) except the fever (P < 0.001). However, the fever was self-limited and could be tolerated well. The application of rAd-p53 through thoracic perfusion for treating MPE had a better efficacy and safety, which could be a potential choice for controlling MPE.

Malignant pleural effusion (MPE) is one of the common complications of lung cancer, which mainly caused by the hyperpermeability of microvascular tissue or invasion of cancer cells into lymphatic vessels^1^. Research show that the incidence of malignant effusion in patients with lung cancer is about 7–23%^2^,^3^ and occurs in 15% of cancer-related deaths^1^, which usually leads to debilitating dyspnoea, worsening quality of life and a poor survival. However, its treatment is quite difficult^2^. The present treatments of MPE included the drainage of pleural effusion, intrapleural chemotherapy and systemic chemotherapy. Unfortunately, not all patients with MPE can benefit from quasi chemotherapy and treatment^1^,^2^,^3^.

The mutation of the p53 gene is one of the most common gene mutations in malignant tumors. It locates on the short arm of human chromosome 17 and encodes a nuclear phosphoprotein^4^. It is reported that the p53 gene is mutated in 50% to 70% of patients with lung cancer^5^, which plays a crucial function in cell cycle regulation, genomic stability, stress induced reaction and DNA repairing^4^. The point mutations on exons 3–9 and 5–8 are mainly common mutations of p53 gene in lung cancer; especially the point mutation on exons 5–8, has been observed in 40–50% of non-small cell lung cancer (NSCLC)^6^. So far, the gene mutation of p53 and overexpression of p53 protein have been investigated to be closely related to the differentiation level, malignant biological behavior and poor prognosis of lung cancer^6^,^7^,^8^. A certain number of studies have been showed that p53 gene transfer has an anti-tumor activity *in vitro* and *in vivo*^5^,^9^,^10^,^11^,^12^,^13^,^14^. Moreover, a series of studies on p53-based gene therapy have been performed from fundamental research (molecule and cell) to clinical applications (human)^1^,^9^,^11^,^12^; for example, a p53-based gene therapy has been approved as part of biological cancer therapy in China^15^.

Recombinant human adenovirus p53 (rAd-p53; Gendicine) applies type 5 adenovirus to carry the exogenetic p53 into malignant tumor cells to express wild type-p53 protein that inhibits the cell division and induces the apoptosis of tumor cells^12^. The rAd-p53 injection (Gendicine) was first developed by SiBiono Gene Technology Co., Ltd, Shenzhen, China, and was approved by the China State Food and Drug Administration (SFDA) for a gene therapy administered intratumorally in 2003^15^. So far, some clinical randomized controlled trials (RCTs) evaluated the efficacy and safety of rAd-p53 by thoracic perfusion in controlling MPE. However, whether or not rAd-p53 has the definite curative effects in treating MPE is still unclear. In addition, its safety still needs to be further evaluated. The aim of this study is to do a systematic evaluation in order to assess the clinical benefits and toxicities of rAd-p53 in treating MPE.

## Results

### Selection and identification of studies

**Figure 1:**
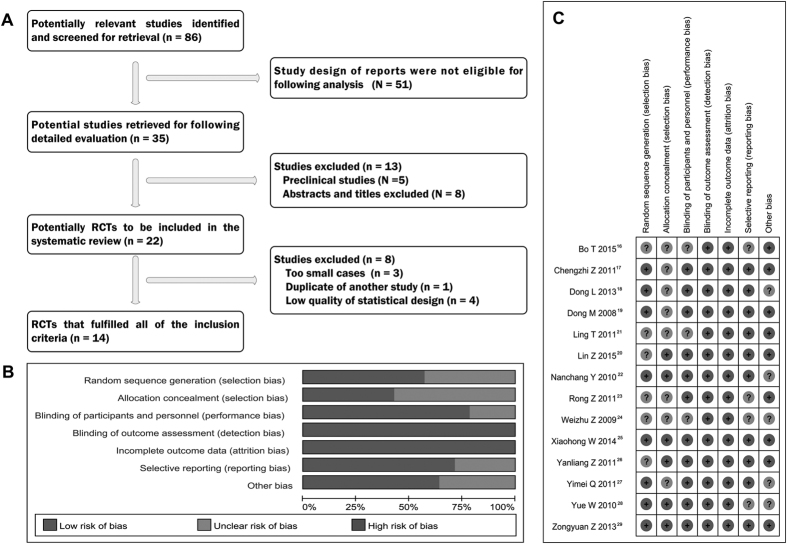
Selection and assessment of literature. (**A**) Studies were retrieved from the electronic bibliographic databases such as PubMed, Embase, Cochrane Library and SCI database. (**B**,**C**) According to the criteria made by the Cochrane Handbook (Version 5.0.1), these results suggested that no heterogeneity existed in eligible RCTs. Overall, these studies had moderate to higher quality.

Originally, we searched 86 relevant reports that talked about the therapy of rAd-p53 in malignant tumors from a series of network databases mentioned above. Through the careful screening, we found 35 studies pertaining to the efficacy and safety of rAd-p53 by thoracic perfusion in treating MPE. However, we had to abandon 13 studies because eight investigations belonged to preclinical studies and five were not the first hand materials (reviews and meeting records). After that, 22 studies were considered as the clinical randomized controlled trials (RCTs) on thoracic perfusion of rAd-p53 for treating MPE. Of them, we abolished eight trials once again because of the following reasons: too small cases (n = 3), duplicate of another study (n = 1) and low quality of statistical design (n = 4). Finally, 14^16^,^17^,^18^,^19^,^20^,^21^,^22^,^23^,^24^,^25^,^26^,^27^,^28^,^29^ studies published between 2008 and 2015 completely fulfilled the inclusion criteria (Fig. 1A).

### Description of included studies

Table 1 listed the detailed study characteristics of 14 studies, which involved in a total of 879 patients, and the patients cases of researches oscillated between 35^24^ and 180^18^ patients. The age of patients ranged from 32 to 82 years old, and the vast majority of MPE were caused by lung cancer, and fewer MPE were from patients with breast cancer^21^,^28^,^29^, digestive cancer^21^, osteosarcoma^21^ and mesothelioma of pleura^28^. All of studies provided the effective endpoint event of observation, which included ORR (overall response rate), DCR (disease control rate), SI (symptom improvement) and AEs (adverse effects). Ten studies designed the thoracic perfusion project of rAd-p53 combined with paclitaxel versus paclitaxel alone for controlling MPE^19^,^20^,^21^,^22^,^23^,^24^,^25^,^26^,^27^,^28^,^29^, one compared the rAd-p53 with Group A streptococcus^17^, and other two assessed the differences between with and without rAd-p53^16^,^19^. The patients of five studies accepted the intravenous chemotherapy besides the therapy of thoracic perfusion at the same time, which included vinorelbine^24^, paclitaxel^27^, gemcitabine^23^, nedaplatin^16^, paclitaxel^20^, these information was shown in Table 1 in detail.

### Study quality of included RCTs and heterogeneity analysis

We assessed the quality of included RCTs according to the criteria shaped by the Cochrane Handbook, which was specialized in evaluating the systematic reviews of Interventions (Version 5.0.1). After assessing the studies, we found ten^17^,^18^,^19^,^20^,^22^,^25^,^26^,^27^,^28^,^29^ of the 14 RCTs (71%) displayed low risk of bias, and remaining four^16^,^21^,^23^,^24^ RCTs showed unclear risk of bias (28.6%) (Table 2 and Fig. 1B,C). The heterogeneity analysis showed that the heterogeneity chi-squared was 3.56 (d.f. = 13) and p = 0.995; also I-squared (variation in OR attributable to heterogeneity) was 0.0%; these results suggested that no heterogeneity existed in included RCTs. Overall, these studies had moderate to higher quality. Therefore, we applied the fixed effects model of meta-analysis to calculate the overall effects.

### Thoracic perfusion of rAd-p53 combined with other agents had a higher ORR compared with other agents alone

**Figure 2:**
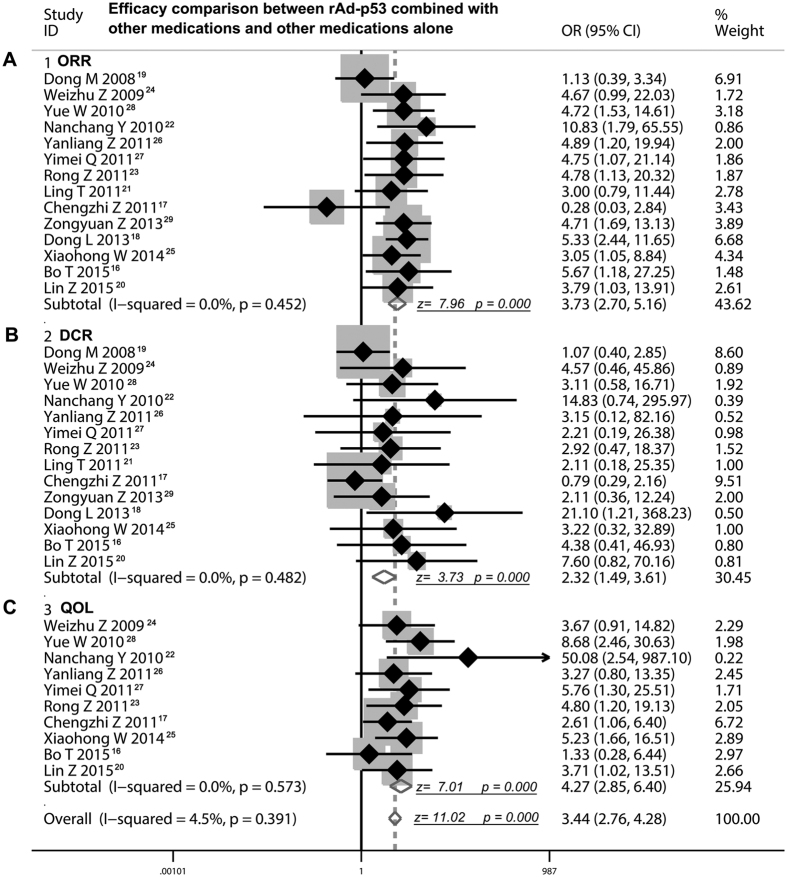
Efficacy comparison of rAd-p53 combined with another agent versus another agent alone by thoracic perfusion for controlling MPE. (**A**) Thoracic perfusion of rAd-p53 combined with other agents had a higher ORR compared with other agents alone. (**B**) Thoracic perfusion of rAd-p53 combined with other agents had a higher DCR compared with other agents alone. (**C**) Thoracic perfusion of rAd-p53 combined with other agents improved the QOL of patients with MPE compared with other agents alone. ORR, overall response rate; DCR, disease control rate; OR, odds ratio; QOL, quality of life.

As shown in Table 3, fourteen RCTs^16^,^17^,^18^,^19^,^20^,^21^,^22^,^23^,^24^,^25^,^26^,^27^,^28^,^29^ recruited in this meta-analysis offered the data on comparison of ORR between rAd-p53 combined other agents and other agents alone by thoracic perfusion for treating MPE. The results from the fixed effects model of meta-analysis displayed that odds ratio was 3.73 (95% CI 2.70 to 5.16; Z value = 7.96, P < 0.001), which indicated that the ORR of rAd-p53 combined other agents was remarkably higher than that of other agents alone (Fig. 2A).

### Thoracic perfusion of rAd-p53 combined with other agents had a higher DCR compared with other agents alone

As shown in Table 3, the data on comparison of overall DCR between rAd-p53 combined other agents and other agents alone by thoracic perfusion for treating MPE was provided by the all included studies^16^,^17^,^18^,^19^,^20^,^21^,^22^,^23^,^24^,^25^,^26^,^27^,^28^,^29^. By the fixed effects model of meta-analysis, we calculated that the odds ratio was 2.32 (95% CI 1.49 to 5.16; Z value = 3.73, P < 0.001), suggesting that therapy of thoracic perfusion of rAd-p53 combined other agents had a far more benefit of DCR than other agents alone (Fig. 2B).

### Thoracic perfusion of rAd-p53 combined with other agents improved the quality of life of patients with MPE compared with other agents alone

Ten of 14 trials^16^,^17^,^20^,^22^,^23^,^24^,^25^,^26^,^27^,^28^ compared the quality of life (QOL) between rAd-p53 combined other agents and other agents alone by thoracic perfusion for treating MPE (Table 3). The results from the fixed effects model of meta-analysis exhibited that odds ratio of two different projects was 4.27 (95% CI 2.85 to 6.40; Z = 7.01, P < 0.001), which demonstrated that the combination therapy of rAd-p53 and other agents remarkably improved the QOL of patients with MPE (Fig. 2C).

### Adverse effects comparison of rAd-p53 combined with other agents versus other agents alone

**Figure 3:**
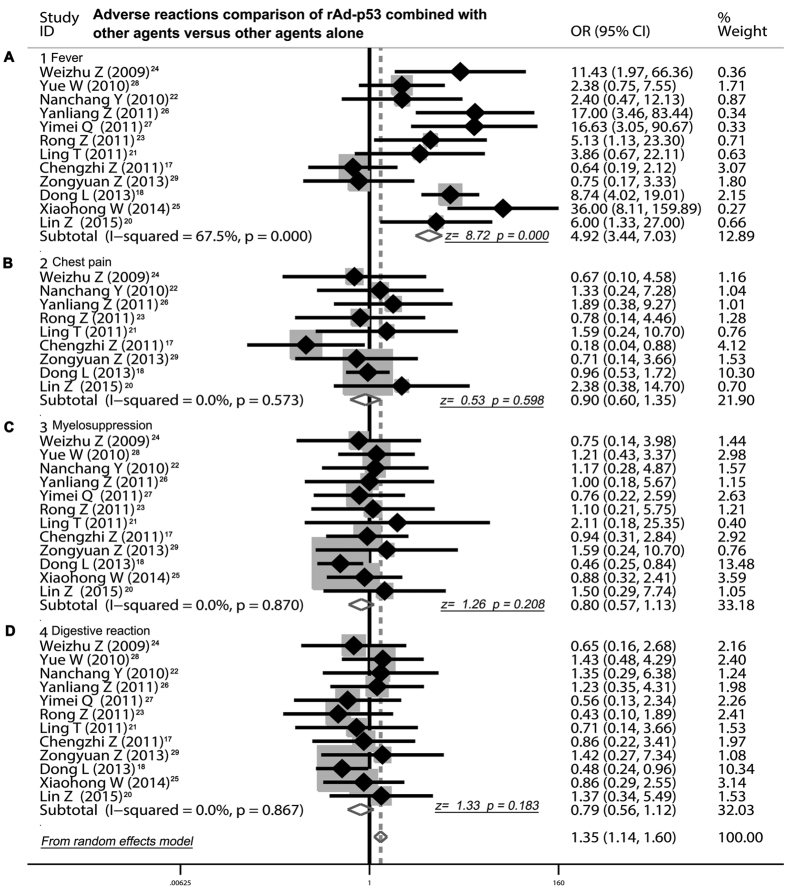
Safety evaluation of rAd-p53 combined with another agent versus another agent alone by thoracic perfusion for controlling MPE. (**A**) The rAd-p53 combination therapy displayed a higher incidence rate of fever than the project of other agents alone. (**B**) The rAd-p53 combined with other agents and other agents alone had a similar incidence of chest pain. (**C**) No difference in incidence rate of myelosuppression was testified between rAd-p53 combined with other agents and other agents alone. (**D**) The digestive reaction of rAd-p53 combination therapy had the same occurrence probability compared with the other agents alone. ORR, overall response rate; DCR, disease control rate; OR, odds ratio.

Table 4 listed all common adverse effects of rAd-p53 combined with other agents versus other agents alone, including fever, chest pain, myelosuppression and digestive reaction. Fourteen trials compared the incidence rate of fever between rAd-p53 combined with other agents and other agents alone^16^,^17^,^18^,^19^,^20^,^21^,^22^,^23^,^24^,^25^,^26^,^27^,^28^,^29^. The rAd-p53 combination therapy displayed a higher incidence rate of fever than the project of other agents alone (OR = 4.92, 95% CI 3.44 to 7.03, P < 0.001) (Fig. 3A). Nine trials^17^,^18^,^20^,^21^,^22^,^23^,^24^,^26^ compared the incidence rate of chest pain and showed that rAd-p53 combined with other agents and other agents alone had a similar incidence of chest pain (OR = 0.90, 95% CI 0. 60 to 1.35, P = 0.598) (Fig. 3B). Results from 14 trials^17^,^18^,^19^,^20^,^21^,^22^,^23^,^24^,^25^,^26^,^27^,^28^,^29^ that were included in our analysis revealed that no difference in incidence rate of myelosuppression (OR = 0.80, 95% CI 0. 57 to 1.13, P = 0.208) (Fig. 3C) was testified between rAd-p53 combined with other agents and other agents alone. Moreover, the digestive reaction of rAd-p53 combination therapy had the same occurrence probability compared with the other agents alone (OR = 0.79, 95% CI 0. 56 to 1.12, P = 0.183) (Fig. 3D).

### Sensitivity and publication bias analysis of included trials

**Figure 4:**
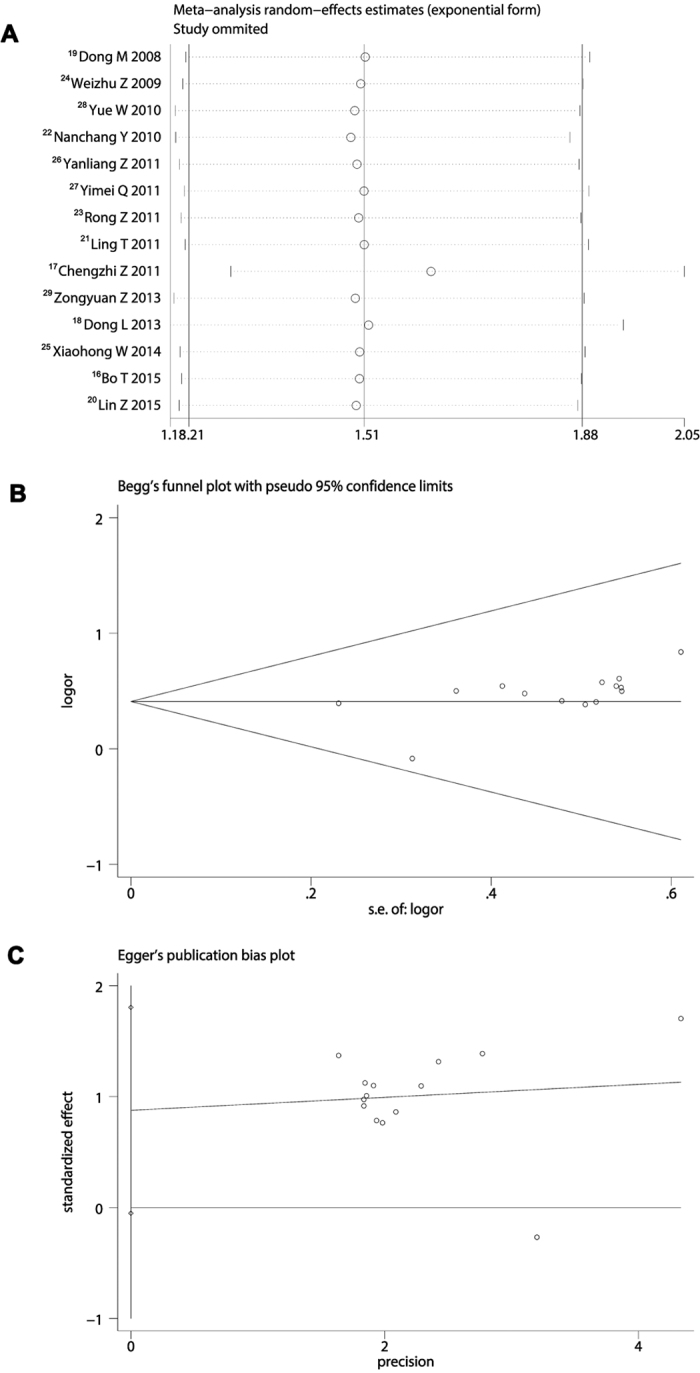
Sensitivity and publication bias analysis. (**A**) Omitting any trial did not shake the pooled effect of meta-analysis. (**B**) Begg’s test showed that P was 0.07, and the funnel plot seems to be nearly symmetrical. (**C**) Egger’s test showed that P value was 0.746, suggesting included trials did not have a potential impact on the pooled effect of present meta-analysis.

Sensitivity analysis of 14 trials clearly revealed that omitting any trial did not shake the pooled effect of meta-analysis (Fig. 4A). Begg’s test showed that Std. Dev. of Score was 18.27 and value of P was 0.07, and the funnel plot seems to be nearly symmetrical (Fig. 4B). Moreover, the result of the Egger’s test was t = 0.33 (P = 0.746) (Fig. 4C). Through comprehensive analysis, we knew that each of 14 included trials did not have a potential impact on the pooled effect of present meta-analysis.

## Discussion

In China, lung cancer has become a highly fatal disease with limited therapeutic options due to aggravation of environmental disruption and the absence of food safety. Researches show various factors affect the prognosis and survival of lung cancer patients. Malignant pleural effusion (MPE), a common complication of lung cancer, has been identified to be a crucial negative factor to remarkably bring down the quality of life (QOL) of patients and reduce the survival^30^. At present, although a number of therapeutic methods are developed to control the MPE, most effective strategies just include local treatment of the chest and platinum based chemotherapy^31^. Today, with the rapid development of genomics and proteomics, various novel targeted drugs based gene mutations have been discovered and developed to cure lung cancer, they have therapeutic selectivity to kill tumor cells but have not significant toxicity to normal cells, and some of them have been used for therapy of MPE^32^,^33^. However, a variety of agents achieve clinical effects to treat MPE, no ideal agent has been identified as having a considerable efficacy. Therefore, the discovery or development of more effective agents and treat strategies has become a major focus in studies investigating possible malignant pleural effusion treatment strategies^31^.

Studying on the relationship between P53 gene and malignant tumors has been disclosed that P53 gene is a tumor suppressing gene, which exerts multiple anti-cancer activity including impeding the cell cycle, promoting apoptosis of tumor cells, suppressing the angiogenesis of tumors^34^. Recombinant adenoviral human p53 gene (rAd-p53) has been shown to be effective for many types of solid malignant tumors^35^. As an important part of the combined therapy for the cancer patients, the p53 gene therapy mediated adenovirus has been blossomed to be a promising treatment strategy^36^. Now, a number of clinical studies about Adp53-based therapies have been reported in many countries and districts. Gendicine is a recombinant human serotype 5 adenovirus in which the E1 region is replaced by a human wild-type p53 expression cassette. Then the adenovirus will carry the exogenetic p53 into malignant tumor cells to express wild type-p53 protein and exert the action in inhibiting the cell division and inducing the apoptosis of tumor cells. Up to now, it has been used in a number of clinical trials for treating various malignant tumors^37^. We searched the relevant studies on the clinical benefits and toxicities of rAd-p53 in treating MPE to disclose the clinical benefits and safety. A total of 14 studies with 879 patients were involved in our analysis. We assessed the quality of included RCTs using the criteria shaped by the Cochrane Handbook and found these studies had a very good clinical homogeneity. Therefore, we applied the fixed effects model of meta-analysis to calculate the overall effects. We made a comparison of overall response rate (ORR) between rAd-p53 combined other agents and other agents alone by thoracic perfusion for treating MPE. The results displayed an odds ratio of 3.73 (95% CI 2.70 to 5.16; P < 0.001), which responded an absolute promotion of 26.4% in ORR, indicating that the rAd-p53 combination had a better benefit on ORR for treating MPE. Moreover, the comparison of overall DCR showed that the odds ratio was 2.32 (95% CI 1.49 to 5.16; P < 0.001), suggesting that therapy of thoracic perfusion of rAd-p53 combined other agents had a far more benefit of DCR than that of other agents alone, which displayed an absolute increase of 7.85% in DCR. Since it is approved in 2003 by the State FDA of China, many RCTs of rAd-p53 have been performed for treating malignant tumors, including lung cancer, liver cancer, malignant glioma and ovarian carcinoma. The results of clinical trials all showed the overall response rates and survival rates were better in the rAd-p53 treatment groups than the control groups^36^.

Health-related quality of life (QOL) has been proposed as an important end-point in studies of outcomes in oncology. Studies of QOL have several benefits when they show evidence that the measurements were conducted and reported appropriately^38^. Indeed, the aim of assessing the impact of disease and treatment on QOL is increasingly stressed as crucial for evaluating the overall treatment effectiveness in cancer clinical trials. Moreover, cancer patients require information not only relate to survival estimates, but also regarding QOL issues^39^. In our analysis, we compared the QOL between rAd-p53 combined other agents and other agents alone by thoracic perfusion for treating MPE, and found that odds ratio of two different projects was 4.27 (95% CI 2.85 to 6.40; Z = 11.02, P < 0.001), suggesting that the combination therapy of rAd-p53 remarkably improved the QOL of patients with MPE. In conclusion, for those patients with advanced MPE, rAd-p53 gene therapy combined with drugs (cisplatin mainly) can enhance their immune function against tumors and it offers a potential better promise of a new and effective therapy. The pleural surface is often invaded by tumor cells, by infusion of rAd-p53 through thoracocentesis and closed drainage into the pleural space, making the exogenetic p53 genes to suppress the growth and proliferation of tumor cells. It seems that adenovirus is a good exogenous p53 gene carrier^31^. The suppression of tumor cells by infusion of rAd-p53 may also have controlled the local and systemic tumor lesions, further improving the QOL of patients with MPE.

In our analysis, more adverse effects (AEs) showed by rAd-p53 combination therapy were fever (46.2%), chest pain (25.6%), myelosuppression (26.3%) and digestive reaction (22.7%). The rAd-p53 combination therapy displayed a higher incidence rate of fever than the project of other agents alone (OR = 4.92, P < 0.001). The application of adenoviral vector has been reported to beget a clear immune response in those who accepted gene therapy, and self-limited fever is always manifested in the process of treatment. Although the fever caused by rAd-p53 is viewed as a obvious adverse effect in clinical treatment, sometimes it also indicates the possibility of efficacy and benefits of rAd-p53 therapy, suggesting that rAd-p53 can effectively mobilize the immune systems of human body to kill the tumor cells^37^. Fortunately, the incidence rate of chest pain, myelosuppression and digestive reaction of rAd-p53 combination therapy had the same occurrence compared with other agents alone (P > 0.05). Get together, though some such common AEs have been reported in the treatment of rAd-p53, no severe AEs have been showed in patients with MPE when accepted the treating with rAd-p53 (Gendicine). Especially, so far no rAd-p53 related fatalities have been clearly reported. In fact, more data have exhibited that the adenovirus-mediated p53 gene therapy could be well tolerated and had a better safety for clinical application.

However, there are some flaws existed in included trials. First, none of included studies focused on the P53 mutation in patients with MPE, so we did not assess whether P53 gene mutation has an influence on the treatment response of Gendicine. But previous studies have shown that Ad-p53 can simultaneously inhibit the growth of human lung adenocarcinoma cell line containing mutant p53 gene and wild-type p53 gene as well. Moreover, Ad-p53 can promote the apoptosis of cancer cells and its anti-tumor effect does not depend on endogenous P53 status^40^,^41^. Second, sample size of some studies is relatively small. Third, all studies were performed in China (because Gendicine was approved by the China State Food and Drug Administration), which may lead to geographical and ethnic differences. In spite of this, these studies still propose a credible suggestion pointing toward that the rAd-p53 (Gendicine) is effective and safe for treating MPE. However, rAd-p53 still needs to be investigated for treating MPE in the future. Anyway, rigorously randomized control trials with large sampler size and multi-centered cooperation should be done before it could be recommended in clinic extensively.

## Conclusions

Thoracic perfusion of rAd-p53 (Gendicine) combined with chemotherapeutic agents has a better benefit of ORR and DCR for treating MPE and improves the QOL of MPE patients, compared with chemotherapeutic agents alone. However, self-limited fever is shown as a special adverse effect for the participation of rAd-p53, but it could be tolerated well. Nevertheless, rigorously randomized control trials should be required before it is used widely.

## Methods and Materials

### Searching and identification of studies

We searched the previous published literature concerning the efficacy and safety of rAd-p53 by thoracic perfusion in treating MPE from the following databases: Medline/PubMed, SpringerLink, Embase, Ovid, Cochrance Library, Web of Science, and CNKI (China National Knowledge Infrastructure). The searching time we defined was from January 2000 to July 2016. The MeSH terms and key words that we defined were as follows: “recombinant human adenovirus p53 injection,” “rAd-p53,” “AD-P53 gene therapy,” “Gendicine,” “malignant pleural effusions,” and “MPE.” We also further searched the relative literature from the reference lists of having been included studies and identified them whether they were available.

### Criteria of inclusion and exclusion for selecting studies

Inclusion criteria: (1) patients who were included in selected studies must be diagnosed with MPE by cytology and histology; (2) study design must be clinical randomized controlled trial comparing rAd-p53 plus another drug to another drug alone; (3) the basic treatment of two groups must be completely equal; (4) the clinical baseline of two groups must be basically equal, such as the scores of life quality, size of MPE, basic laboratory index and vital signs; (5) the rAd-p53 and another drug must be given through thoracic perfusion; and (6) included studies must have reported the outcome measures, which included response, (RR), disease control rate (DCR), symptom improvement (SI) and adverse effects (AEs). Exclusion criteria: (1) non-original articles, such as abstract, meeting record, editorial, review and correspondence; (2) non-human studies; (3) used excessive other adjutant drugs; (4) the funding and expenditure of studies were provided by the producer of rAd-p53; (5) control design was not balanced; (6) lost of follow up rate of patients was above 15%; and (7) the study quality of the literature is too low when assessed by the evaluation criteria from the Cochrane Handbook (Version 5.0.1); and (8) lack of ethics statement.

### Extraction of study variables

The variables we shaped included: (1) authors of study, years of publication, the number of patients who participated each RCT; (2) gender and histology of lung cancer patients; (3) the quality of life of patients; (4) the specific process of clinical intervention; (5) RR and DCR of clinical intervention; and (6) improvement of SI and AEs.

### Supervision of clinical intervention

Trial design: clinical RCTs of rAd-p53 combined with another agent versus another agent alone by thoracic perfusion for controlling MPE. Type of clinical interventions: the dosage of rAd-p53 was defined in accordance with the instruction of producer and the frequency of dosing at least two times. Indicators of efficacy that evaluated rAd-p53 included the first outcome ORR and DCR, and secondary quality of life (QOL) and AEs.

### Quality assessment of included studies

The Cochrane Handbook (Version 5.1.0) shaped the criteria for evaluating the clinical randomized controlled trials. We acted in accordance with the criteria to assess the quality of included studies. The criteria included the following aspects: (1) random sequence generation of patients; (2) setting blinding; (3) allocation concealment; (4) outcome data; (5) reporting of selective outcome; (6) other bias factors; and 7) intention-to-treat (ITT).

### Statistics process

We employed two different statistical models, fixed effects model and random effects model, to measure the safety and efficacy of rAd-p53 thoracic perfusion for treating MPE. We utilized the chi-square test and I^2^ value to determine whether the heterogeneity existed in included studies. If no heterogeneity existed, the method of fixed effects model was adopted, or using the random effects model. Relevant variables were calculated by the statistics estimation of odds ratios (OR) and a calculated 95% confidence interval (CI). The pooled effect was measured through a value of Z-scores and P < 0.05 was considered as having a significance of statistics. We also performed a sensitivity analysis of meta-analysis, which omitted each study one by one in order to know clearly whether any study could shake the pooled effect of meta-analysis. Further, we drew a funnel plot of included studies, and conducted the Begg’s test and Egger’s test to evaluate the publication biases. We used the following statistics software to do all statistics process: SPSS (version 19.0, Chicago, USA), Stata 13.0 (StataCorp LP, USA) and RevMan 5.2 (Cochrane Collaboration). We defined the p-value was two-sided, and P value less than 0.05 indicated a statistical significance.

## Additional Information

**How to cite this article**: Biaoxue, R. *et al*. Evaluation of efficacy and safety for recombinant human adenovirus-p53 in the control of the malignant pleural effusions via thoracic perfusion. *Sci. Rep.* **6**, 39355; doi: 10.1038/srep39355 (2016).

**Publisher’s note:** Springer Nature remains neutral with regard to jurisdictional claims in published maps and institutional affiliations.

## Figures and Tables

**Table 1:** Patient characteristics of the clinical trials reviewed.

Study	N	M/F	Age (Years)	Sources of tumor (N)	Histology of Lung cancer LAC/LSCC/SCLC/Other	Quality of Life	End point
Dong M^19^	48	29/19	35–82	MPE (48)	NA	KPS ≥ 60	RR, DCR, SI
Weizhu Z^24^	35	19/16	37–75	Lung cancer (35)	19/14/0/2	KPS ≥ 60	RR, DCR, SI, AEs
Yue W^28^	95	50/45	34–75	Lung cancer (46) Breast cancer (21) Mesothelioma of pleura (9) Others (19)	NA	KPS ≥ 60	RR, DCR, SI, AEs
Nanchang Y^22^	46	24/22	40–70	NSCLC (46)	20/18/0/8	KPS ≥ 60	RR, DCR, SI, AEs
Yimei Q^27^	43	25/18	38–75	Lung cancer (43)	24/15/0/4	KPS ≥ 60	RR, DCR, SI, AEs
Yanliang Z^26^	40	23/17	34–75	Lung cancer (40)	31/9	ZPS ≤ 2	RR, DCR, SI, AEs
Rong Z^23^	39	21/17	37–45	Lung cancer (39)	17/20/0/2	KPS ≥ 30	RR, DCR, SI, AEs
Ling T^21^	40	24/16	39–73	Lung cancer (29) Breast cancer (5) Digestive cancer (4) Osteosarcoma (1)	NA	KPS ≥ 60	RR, DCR, SI, AEs
Chengzhi Z^17^	96	67/29	29–77	Lung cancer (96)	71/12/13	ZPS ≤ 2	RR, DCR, SI, AEs
Zongyuan Z^29^	80	44/36	32–65	Lung cancer (48) Breast cancer (32)	NA	NA	RR, DCR, AEs
Dong L^18^	180	119/61	61.4 ± 9.1	Lung cancer (180)	108/45/27	NA	RR, DCR, SI, AEs
Xiaohong W^25^	60	43/17	38–73	NSCLC (60)	NA	KPS ≥ 70	RR, DCR, SI, AEs
Bo T^16^	36	NA	NA	Lung cancer (36)	NA	NA	RR, DCR, SI, AEs
Lin Z^20^	41	24/17	69.2 ± 10.2	NSCLC (41)	8/20/0/13	KPS ≥ 60	RR, DCR, SI, AEs

N = number of patients; M/F = male/female; LAC, lung adenocarcinoma; LSCC, lung squamous cell carcinoma; SCLC, small cell lung cancer; MPE = malignant pleural effusion; NA = not available; KPS, Karnofsky performance scale index; ZPS, performance scale index made by Eastern Cooperative Oncology Group; RR = response rate; DCR = disease control rate; SI = symptom improvement; AEs = adverse effects.

**Table 2:** Raw data and methodological quality of included trials.

Studies	Region	Sequence generation	Allocation concealment	Blind	Outcome data	Selective outcome reporting	Other sources of bias	ITT	Risk of bias
Dong M^19^	Single center	Random number table (SPSS)	Insufficient	Clear	Yes	No	Unclear	Yes	Low risk of bias
Weizhu Z^24^	Single center	NA	Insufficient	Unclear	Yes	No	Unclear	Unclear	Unclear risk of bias
Yue W 2010^22^	Single center	Random number table (SAS)	Clear	Clear	Yes	No	Unclear	Yes	Low risk of bias
Nanchang Y^22^	Single center	Random number table (SAS)	Clear	Clear	Yes	No	Unclear	Yes	Low risk of bias
Yanliang Z^26^	Single center	Random number table (SPSS)	Insufficient	Clear	Yes	No	Unclear	Yes	Low risk of bias
Yimei Q^27^	Single center	Random number table (SPSS)	Insufficient	Clear	Yes	No	Unclear	Yes	Low risk of bias
Rong Z^23^	Single center	NA	Insufficient	Unclear	Yes	No	Unclear	Unclear	Unclear risk of bias
Ling T^21^	Single center	NA	Insufficient	Unclear	Yes	No	Unclear	Unclear	Unclear risk of bias
Chengzhi Z^17^	Single center	Random number table (SPSS)	Insufficient	Clear	Yes	No	Unclear	Yes	Low risk of bias
Zongyuan Z^29^	Single center	Random number table (SAS)	Clear	Clear	Yes	No	Unclear	Yes	Low risk of bias
Dong L^18^	Single center	Random number table (SPSS)	Insufficient	Clear	Yes	No	Unclear	Yes	Low risk of bias
Xiaohong W^25^	Single center	Random number table (SPSS)	Clear	Clear	Yes	No	Unclear	Yes	Low risk of bias
Bo T^16^	Single center	NA	Insufficient	Unclear	Yes	Unclear	Unclear	Unclear	Unclear risk of bias
Lin Z^20^	Single center	Random number table (SPSS)	Insufficient	Clear	Yes	No	Unclear	Yes	Low risk of bias

ITT, intention-to-treat.

**Table 3:** Efficacy of recombinant human Ad- p53 injection in treating malignant pleural effusion.

Study			Intravenous chemotherapy simultaneously			Efficacy of therapy									
	Study design (N)			Pleural perfusion (N)		Group 1				Group 2				Improvement of SI (N, %)	
	Group 1	Group 2		Group 1	Group 2	CR	PR	SD	PD	CR	PR	SD	PD	Group 1	Group 2
Dong M^19^	27	21	No	rAd-p53 + P	P	6	11	4	6	4	5	8	4	NA	NA
Weizhu Z^24^	17	18	Vinorelbine	rAd-p53 + P	P	8	6	2	1	6	3	5	4	11	6
Yue W 2010^22^	31	32	No	rAd-p53 + P	P	2	23	4	2	0	15	13	4	27	14
Nanchang Y^22^	15	16	No	rAd-p53 + P	P	3	10	2	1	1	5	5	4	15	6
Yanliang Z^26^	20	20	No	rAd-p53 + P	P	4	12	4		2	7	11		16	11
Yimei Q^27^	22	21	Paclitaxel	rAd-p53 + P	P	11	8	2	1	9	3	7	2	19	11
Rong Z^23^	21	17	Gemcitabine	rAd-p53 + P	P	6	11	2	2	2	6	5	4	14	5
Ling T^21^	20	20	No	rAd-p53 + P	P	2	13	4	1	1	9	8	2	NA	NA
Chengzhi Z^17^	46	50	GP	rAd-p53	Group A streptococcus	15.	18	3	10	18	21	2	9	36	29
Zongyuan Z^29^	40	40	No	rAd-p53 + P	P	5	28	5	2	2	18	16	4	NA	NA
Dong L^18^	90	90	TP	rAd-p53	None	34	46	10	0	10	44	27	9	NA	NA
Xiaohong W^25^	30	30	DP	rAd-p53 + P	P	3	18	8	1	2	11	14	3	24	13
Bo T^16^	20	16	Nedaplatin	rAd-p53	None	10	7	2	1	4	4	5	3	16	12
Lin Z^20^	20	21	Paclitaxel	rAd-p53 + P	P	6	8	5	1	2	6	7	6	13	7

N = cases; rAd-p53 = recombinant human Ad- p53 injection; Group 1 = recombinant human Ad- p53 injection combined with other therapy; Group 2 = other therapy alone; CR = complete response; PR = partial response; SD = stable disease; PD = progressive disease; P = cisplatin; TP = Docetaxel + cisplatin; GP = Gemcitabine + cisplatin; NA = not available.

**Table 4:** Comparison of adverse events between rAd-p53 with other therapy and other therapy alone.

Study	Fever (N)		Chest pain (N)		Myelosuppression (N)		Digestive reaction (N)	
	Group 1	Group 2	Group 1	Group 2	Group 1	Group 2	Group 1	Group 2
Weizhu Z^24^	10/17	2/18	2/17	3/18	3/17	4/18	5/17	7/18
Yue W 2010^22^	11/31	6/32	‒	‒	12/31	11/32	10/31	8/32
Nanchang Y^22^	6/16	3/15	4/16	3/15	7/16	6/15	6/16	4/15
Yanliang Z^26^	15/20	3/20	5/20	3/20	3/20	3/20	9/20	8/20
Yimei Q^27^	14/22	2/21			8/22	9/21	4/22	6/21
Rong Z^23^	11/21	3/17	3/21	3/17	4/21	3/17	4/21	6/17
Ling T^21^	6/20	2/20	3/20	2/20	2/20	1/20	3/20	4/20
Chengzhi Z^17^	5/46	8/50	2/46	10/50	7/46	8/50	4/46	5/50
Zongyuan Z^29^	4/20	5/20	3/20	4/20	3/20	2/20	4/20	3/20
Dong L^18^	47/90	10/90	42/90	43/90	25/90	38/90	16/90	28/90
Xiaohong W^25^	24/30	3/30	‒	‒	15/30	16/30	9/30	10/30
Lin Z^20^	10/20	3/21	4/20	2/21	4/20	3/21	6/20	5/21
	P < 0.05		P > 0.05		P > 0.05		P > 0.05	

Group 1 = recombinant human Ad- p53 injection combined with other therapy; Group 2 = other therapy alone.
